# Rapid Increase in Circulation of the SARS-CoV-2 B.1.617.2 (Delta) Variant — Mesa County, Colorado, April–June 2021

**DOI:** 10.15585/mmwr.mm7032e2

**Published:** 2021-08-13

**Authors:** Rachel Herlihy, Wendy Bamberg, Alexis Burakoff, Nisha Alden, Rachel Severson, Eric Bush, Breanna Kawasaki, Brynn Berger, Elizabeth Austin, Meghan Shea, Eduardo Gabrieloff, Shannon Matzinger, April Burdorf, Janell Nichols, Kim Goode, Alana Cilwick, Chelsea Stacy, Erin Staples, Ginger Stringer

**Affiliations:** ^1^Colorado Department of Public Health and Environment; ^2^Mesa County Public Health Department, Grand Junction, Colorado; ^3^CDC COVID-19 Response Team.

On May 5, 2021, the Colorado Department of Public Health and Environment (CDPHE) identified the first five COVID-19 cases caused by the SARS-CoV-2 B.1.617.2 (Delta) variant in Mesa County in western Colorado (population 154,933, <3% of the state population). All five initial cases were associated with school settings. Through early June, Mesa County experienced a marked increase in the proportion of Delta variant cases identified through sequencing: the 7-day proportion of sequenced specimens identified as B.1.617.2 in Mesa County more than doubled, from 43% for the week ending May 1 to 88% for the week ending June 5. As of June 6, more than one half (51%) of sequenced B.1.617.2 specimens in Colorado were from Mesa County. CDPHE assessed data from surveillance, vaccination, laboratory, and hospital sources to describe the preliminary epidemiology of the Delta variant and calculate crude vaccine effectiveness (VE). Vaccination coverage in early May in Mesa County was lower (36% of eligible residents fully vaccinated) than that in the rest of the state (44%). Compared with that in all other Colorado counties, incidence, intensive care unit (ICU) admissions, and COVID-19 case fatality ratios were significantly higher in Mesa County during the analysis period, April 27–June 6, 2021. In addition, during the same time period, the proportion of COVID-19 cases in persons who were fully vaccinated (vaccine breakthrough cases) was significantly higher in Mesa County compared with that in all other Colorado counties. Estimated crude VE against reported symptomatic infection for a 2-week period ending June 5 was 78% (95% confidence interval [CI] = 71%–84%) for Mesa County and 89% (95% CI = 88%–91%) for other Colorado counties. Vaccination is a critical strategy for preventing infection, serious illness, and death from COVID-19. Enhanced mitigation strategies, including masking in indoor settings irrespective of vaccination status, should be considered in areas with substantial or high case rates.

Whole genome sequencing is performed in the CDPHE laboratory on specimens submitted as part of sentinel surveillance (38 sites across Colorado, including one acute care hospital in Mesa County), as well as for cluster and outbreak response and on suspected variants (reverse transcription–polymerase chain reaction [RT-PCR]–positive specimens with S-gene target failure associated with the B.1.1.7 lineage) (*1*). The Colorado Electronic Disease Reporting System (CEDRS), a surveillance system managed by CDPHE, was used to identify reported confirmed or probable cases of COVID-19 occurring from April 27, the date of illness onset for the first Delta variant case in Mesa County, to June 6, when sequencing identified B.1.617.2 as the dominant variant in Colorado (*2*). The Colorado Immunization Information System (CIIS) was used to verify COVID-19 vaccination status; vaccine breakthrough infections were identified using personally identifying information to match cases in CEDRS to CIIS entries* (*3*). Crude VE against reported symptomatic infection was estimated and compared among Mesa County and all other Colorado counties using a screening method outlined by the World Health Organization^†^ as a rapid tool to assess whether a vaccine is performing as expected (*4*). To better determine settings where the Delta variant was spreading, outbreak data during April 22–June 26 were obtained from the CDPHE outbreak database, which contains information on all reported COVID-19 outbreaks in Colorado and outbreak line lists.^§^ Residential care facility vaccination data were obtained from EMResource, a capacity planning tool used by CDPHE for facility-level reporting of aggregate COVID-19 vaccinations. Incidence of SARS-CoV-2 infection and proportions of outcomes and vaccination rates among patients living in Mesa County and all other Colorado counties were compared and p-values were calculated using chi-square or Fisher’s exact tests. This activity was reviewed by CDC and was conducted consistent with applicable federal law and CDC policy.^¶^

During April 27–June 6, a total of 1,945 COVID-19 cases were reported in Mesa County through CEDRS (incidence = 1,255 per 100,000). Compared with that in all other Colorado counties, incidence, overall ICU admissions, and overall case fatality ratios were significantly higher in Mesa County (Table). In addition, the proportion of breakthrough cases was significantly higher in Mesa County than in all other Colorado counties. In Mesa County, the proportion of persons aged ≥65 years with COVID-19 who were fully vaccinated (27.5%) was significantly higher than that in all other Colorado counties (17.4%). The crude VE against reported symptomatic infection for a 2-week period ending June 5 was 78% (95% CI = 71%–84%) for Mesa County and 89% (95% CI = 88%–91%) for all other Colorado counties.**

**TABLE:** Age-specific incidence, clinical outcomes, and vaccination status among COVID-19 cases in Mesa and other counties — Colorado, April 27–June 6, 2021

Characteristic	Mesa County	Other Colorado counties	p-value^†^
**Total COVID-19 cases, no.**	**1,945**	**35,494**	—
**Age group, yrs**			
0–17	477	7,603	—
18–64	1,246	25,466	—
≥65	222	2,425	—
Overall incidence*	1,255	633	<0.001
**Age group, yrs**			
0–17	1,408	620	<0.001
18–64	1,377	714	<0.001
≥65	726	297	<0.001
**Hospital admission, no./No. (%)**	142/1,945 (7.3)	2,448/35,494 (6.9)	0.49
**Age group, yrs**			
0–17	3/477 (0.6)	97/7,603 (1.3)	0.22
18–64	69/1,246 (5.5)	1,554/25,466 (6.1)	0.42
≥65	70/222 (31.5)	797/2,425 (32.9)	0.69
**ICU admission among hospitalized patients, no./No. (%)**	49/142 (34.5)	583/2,448 (23.8)	0.004
**Age group, yrs**			
0–17	1/3 (33.3)	17/97 (17.5)	0.45
18–64	25/69 (36.2)	356/1,554 (22.9)	0.01
≥65	23/70 (32.9)	210/797 (26.4)	0.24
**Overall CFR, no./No. (%)**	29/1,945 (1.5)	299/35,494 (0.8)	0.003
**Age group, yrs**			
0–17	1/477 (0.2)	2/7,603 (0.03)	0.16
18–64	7/1,246 (0.6)	101/25,466 (0.4)	0.37
≥65	21/222 (9.5)	196/2,425 (8.1)	0.47
**CFR, hospitalized patients, no./No. (%)**	22/142 (15.5)	198/2,448 (8.1)	0.002
**Age group, yrs**			
0–17	1/3 (33.3)	1/97(1.0)	0.06
18–64	5/69 (7.2)	55/1,554 (3.5)	0.11
≥65	16/70 (22.9)	142/797 (17.8)	0.29
**Fully vaccinated^§,^**^¶^, **no./No. (%)**	136/1,945 (7.0)	1,715/35,397 (4.8)	<0.001
**Age group, yrs**			
0–17	2/477 (0.4)	10/7,591 (0.1)	0.16
18–64	73/1,246 (5.9)	1,283/25,381 (5.1)	0.21
≥65	61/222 (27.5)	422/2,425 (17.4)	<0.001

**Abbreviations:** CFR = case fatality ratio; ICU = intensive care unit.

* Cases per 100,000 population.

^†^ Calculated using chi-square test or Fisher’s exact test.

^§^ A fully vaccinated person is one who has completed all recommended doses of a Food and Drug Administration–authorized COVID-19 vaccine, including Pfizer-BioNTech, Moderna, and Janssen (Johnson & Johnson) ≥14 days before a positive SARS-Co-V-2 test result.

^¶^ Vaccination status was missing for 97 persons.

Among 18,475 sequenced specimen results reported in Colorado through June 6, a total of 783 infections with the Delta variant were identified; more than one half (400; 51.1%) of these occurred among Mesa County residents, even though the county accounts for <3% of the state’s population. Symptomatic illness was reported in 304 (76.0%) of the 400 Delta variant infections in Mesa County residents and 251 (65.5%) of 383 Delta variant infections in other counties. The 7-day percentage of sequenced sentinel specimens identified as SARS-CoV-2 B.1.617.2 in Mesa County increased from 43% for the week ending May 1 to 88% for the week ending June 5 (Figure). During the 5-week period, 67% (51 of 76) of sentinel surveillance specimens in Mesa County were identified as B.1.617.2 compared with 15% (248 of 1,637) of specimens from all other Colorado counties sequenced over the same time frame.

**FIGURE:**
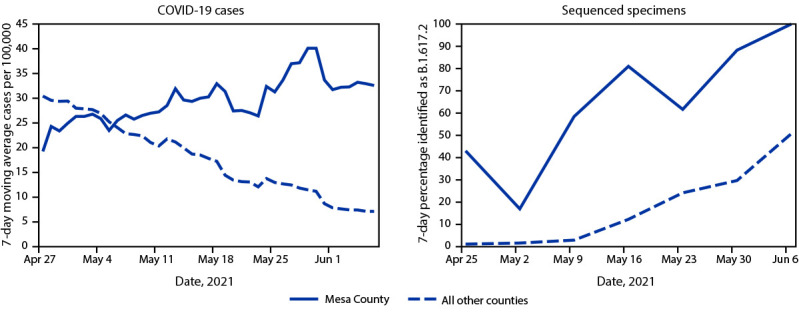
Number of COVID-19 cases and proportion of B.1.617.2 (Delta) variant infections in Mesa and other counties —Colorado, April 27–June 6, 2021

During April 22–June 26, a total of 37 COVID-19 outbreaks were reported in Mesa County; 13 (35%) in residential care facilities, 11 (30%) in schools, two (5%) in correctional facilities, and 11 (30%) in other settings. Twelve outbreaks, including seven in residential care facilities, had at least one Delta variant case. Average vaccination coverage in these seven residential facilities was 87% among residents (range = 50%–97%) and 50% among staff members (range = 6%–69%); attack rates among residents ranged from 0% to 54.6% (median = 1.2%) and among staff members from 2.2% to 25.5% (median = 10.0%). Five of these seven outbreaks involved at least one case in a fully vaccinated resident or staff member.^††^

## Discussion

The Delta variant is highly transmissible; within 5 weeks of first identification, the Delta variant became the dominant SARS-CoV-2 variant in Mesa County, Colorado and is also now the predominant variant in the United States (*5*). Higher ICU admissions and case fatality ratios in Mesa County compared with those in the rest of the state are consistent with previous reports that infections with the Delta variant might result in more severe outcomes (*6*,*7*). The slightly lower crude VE estimate against symptomatic infection in Mesa County may lend support to previous findings that COVID-19 vaccines provide modestly lower protection against symptomatic infection with the Delta variant (*8*). Alternatively, because the Delta variant was circulating at higher levels in Mesa County than in other Colorado counties, the lower VE in Mesa County might reflect the much higher exposure to circulating virus among vaccinated persons.

The findings in this report are subject to at least four limitations. First, lack of genetic sequencing for all SARS-CoV-2 isolates likely affected estimated rates and proportions; the number of outbreaks involving the Delta variant might be underreported for this reason. Second, sentinel surveillance might not provide a fully representative sample of sequence types in Colorado because the specimens originate from hospitals and likely include more specimens from inpatients and emergency department patients compared with specimens from other testing sites. Third, the screening method provides rapid crude VE estimates that do not control for possible effects of confounding or clustering. Some of the differences between VE and severity of illness in Mesa County and that in other counties might be due to differences in the age distribution of patients and the inclusion of cases associated with outbreaks in congregate settings. However, CDPHE estimates that fewer than 10% of cases during the time period occurred in congregate settings. Finally, differences in vaccination coverage in some of these populations might be an additional confounding factor when estimating crude VE at the county and state levels. VE studies with more rigorous methods and the power to estimate protection against severe outcomes are needed to better understand the potential impact of the Delta variant.

Vaccination is a critical strategy for preventing infection, serious illness, and death associated with SARS-CoV-2 (including the Delta variant). Additional targeted prevention strategies (e.g., masking in indoor settings irrespective of vaccination status) and adherence to prevention strategies (e.g., surveillance testing and infection prevention and control procedures) are prudent in areas with high circulation of the Delta variant and in higher risk settings, such as residential care facilities.

**Summary**

**What is already known about this topic?**

The highly transmissible B.1.617.2 (Delta) variant of SARS-CoV-2 has become the predominant circulating U.S. strain.

**What is added by this report?**

During April–June 2021, COVID-19 cases caused by the Delta variant increased rapidly in Mesa County, Colorado. Compared with that in other Colorado counties, incidence, intensive care unit admissions, COVID-19 case fatality ratios, and the proportion of cases in fully vaccinated persons were significantly higher in Mesa County. Crude vaccine effectiveness against symptomatic infection was estimated to be 78% for Mesa County and 89% for other Colorado counties.

**What are the implications for public health practice?**

Vaccination is critical for preventing infection, serious illness, and death associated with SARS-CoV-2 infection (including the Delta variant). Multicomponent prevention strategies, such as masking in indoor settings irrespective of vaccination status as well as optimal surveillance testing and infection prevention and control, should be considered in areas of high incidence.

## Footnotes

* SARS-CoV-2 RNA or antigen in a respiratory specimen collected from a person ≥14 days after they have completed all recommended doses of the primary series for a Food and Drug Administration–authorized COVID-19 vaccine.

† Crude VE was estimated as (1-[{PCV/(1-PCV)}/{PPV/(1-PPV)}]) following World Health Organization interim guidance on conducting VE evaluations in the setting of new SARS-CoV-2 variants where PCV is the observed percentage of cases in persons who are vaccinated and PPV is the percentage of a comparable group in the population who are vaccinated. The PPV used in the calculations for Mesa County and other Colorado counties was from May 7, 2021, approximately 2 weeks before the anticipated onset for cases included in the PCV estimate. PPV included only vaccine-eligible persons and PCV was limited to symptomatic persons who were vaccine-eligible.

§ An outbreak in a residential care facility (skilled nursing facility, assisted living residence, intermediate care facility, or group home) is defined as the occurrence of two or more confirmed cases of COVID-19 among residents and staff members in a facility within 14 days, or one confirmed case and two or more probable cases of COVID-19 among residents and staff members in a facility within 14 days. Until May 31, 2021, the definition of a school outbreak was defined as two or more confirmed COVID-19 cases among students, teachers, and staff members from separate households within 14 days in a single classroom, cohort, or activity or other close contact in the school setting; or one confirmed case and two or more probable cases of COVID-19 among students, teachers, and staff members from separate households within 14 days in a single classroom, cohort, or activity or other close contact in the school setting. Starting June 1, the definition changed from two or more to five or more cases of COVID-19, of which at least one patient has had a positive molecular amplification test or antigen test, among students, teachers, and staff members from separate households within 14 days in a single classroom, cohort, or activity or other close contact in the school setting.

¶ 45 C.F.R. part 46, 21 C.F.R. part 56; 42 U.S.C. Sect. 241(d); 5 U.S.C. Sect. 552a; 44 U.S.C. Sect. 3501 et seq.

** For Mesa County, PPV was 36.2% and PCV was 11.0%. For other Colorado counties, PPV was 44.2% and PCV was 7.9%.

†† A fully vaccinated person is one who has completed all recommended doses of an FDA–authorized COVID-19 vaccine, including Pfizer-BioNTech, Moderna, and Janssen (Johnson & Johnson) ≥14 days before a positive SARS-CoV-2 test result.
